# Simulation of Contrast Agent Transport in Arteries with Multilayer Arterial Wall: Impact of Arterial Transmural Transport on the Bolus Delay and Dispersion

**DOI:** 10.1155/2014/803276

**Published:** 2014-11-17

**Authors:** Min Xu, Xiao Liu, Ang Li, Yubo Fan, Anqiang Sun, Xiaoyan Deng, Deyu Li

**Affiliations:** Key Laboratory for Biomechanics and Mechanobiology of the Ministry of Education, School of Biological Science and Medical Engineering, Beihang University, Beijing 100191, China

## Abstract

One assumption of DSC-MRI is that the injected contrast agent is kept totally intravascular and the arterial wall is impermeable to contrast agent. The assumption is unreal for such small contrast agent as Gd-DTPA can leak into the arterial wall. To investigate whether the unreal assumption is valid for the estimation of the delay and dispersion of the contrast agent bolus, we simulated flow and Gd-DTPA transport in a model with multilayer arterial wall and analyzed the bolus delay and dispersion qualified by mean vascular transit time (MVTT) and the variance of the vascular transport function. Factors that may affect Gd-DTPA transport hence the delay and dispersion were further investigated, such as integrity of endothelium and disturbed flow. The results revealed that arterial transmural transport would slightly affect MVTT and moderately increase the variance. In addition, although the integrity of endothelium can significantly affect the accumulation of contrast agent in the arterial wall, it had small effects on the bolus delay and dispersion. However, the disturbed flow would significantly increase both MVTT and the variance. In conclusion, arterial transmural transport may have a small effect on the bolus delay and dispersion when compared to the flow pattern in the artery.

## 1. Introduction

Dynamic susceptibility contrast magnetic resonance imaging (DSC-MRI) has been shown to be a powerful technique to qualify cerebral blood flow and is playing an increasing role in diagnosis of acute ischemic stroke [1, 2]. However, the accuracy of perfusion parameters obtained from DSC-MRI is challenged by the delay and dispersion of the contrast agent bolus caused by the estimated arterial input function (AIF) [3, 4].

Cerebral blood flow quantification requires knowledge of the arterial input function that is the concentration of contrast agent in the feeding vessel to the tissue of interest. In theory the AIF should be measured at the feeding vessel close to the tissue of interest [5, 6]. However, due to technical difficulties, it is commonly estimated from a major artery (e.g., the middle cerebral artery) for analysis of cerebral perfusion [5]. Delay and dispersion of the contrast agent may occur between the position of the AIF estimated and the tissue of interest, which would lead to underestimation of the blood flow [5].

Another implicit assumption of the technique of DSC-MRI related to the estimation of AIF is that no or a negligible amount contrast agent penetrates the arterial wall, due to the fact that, in general, the vascular wall is considered irrelevant for the contrast mechanism in DSC-MRI. The assumption is tenable for the intact blood brain barrier (BBB) [7]. However, for a damaged BBB which usually occurs in the stroke and normal arterial wall except in the brain, the assumption is unreal. The commonly used contrast agent gadolinium diethylenetriominepentax-acetic acid (Gd-DTPA) with hydrodynamic diameter of less than 2 nm can easily leak into the arterial wall, since it has been demonstrated that the hydrophilic solute with the diameter less than 7.0 nm can be transported through the intercellular junction of endothelial cell into arterial wall [8]. Whether the unreal assumption is valid for the estimation of the delay and dispersion of the contrast agent bolus has never been verified. It seems that the leakage of contrast agent into the arterial wall may affect contrast agent transport, which might cause distortion of the contrast concentration-time curve and hence the delay and dispersion of the contrast agent bolus.

To investigate the delay and dispersion effects of a bolus of contrast agent, two approaches were usually applied. One that has been commonly used was convolving the estimated AIF with a vascular transport function (VTF). The VTFs were quite differently assumed, ranging from the simple model of a single-exponential to the more sophisticated model of a feeding artery in series with small parallel vessels [3, 9]. The disadvantage of the method is that accuracy of the different VTF models is hard to assess. The other was the simulation of the blood flow and contrast agent transport using computational fluid dynamics (CFD) to describe the delay and dispersion errors of the contrast agent bolus [10–12]. The approach has been used to investigate the effect of carotid artery stenosis on the cerebral blood flow quantification and to simulate the dispersion along a simplified coronary artery with different stenosis under steady and unsteady flow condition. Although these studies indicate that CFD simulations were useful to investigate the delay and dispersion of the contrast agent bolus, all the studies were based on the unverified assumption of impermeable arterial wall.

In the present study, to investigate whether the unreal basic assumption of DSC-MRI is valid for the estimation of the delay and dispersion of the contrast agent bolus, we formulated the arterial wall as a five-layer model and numerically simulated the flow and the transport of contrast agent in the model using CFD. This five-layer model included the endothelial glycocalyx layer (EGL), the endothelium, the intima, the internal elastic lamina (IEL), and the media, which were all treated as macroscopically homogeneous porous media. The effects of different factors that may affect contrast agent transport such as the integrity of endothelium and the disturbed flow after the stenosis of artery on the delay and dispersion of contrast agent were further analyzed.

## 2. Methods

### 2.1. Geometry of the Model

**Figure 1:**
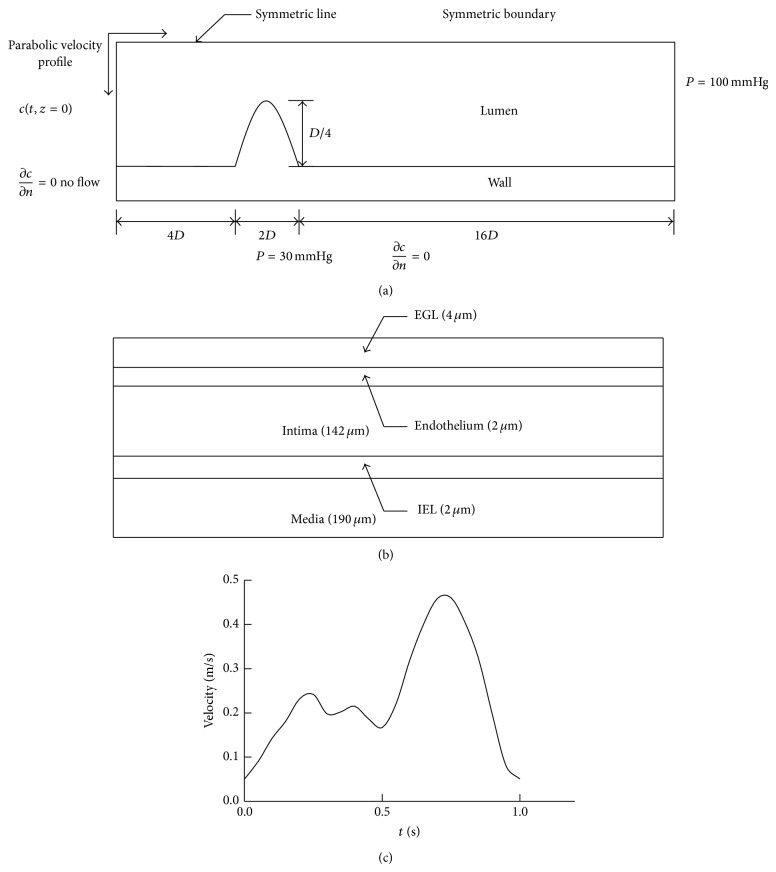
Schematic illustration of the computational geometry and boundary conditions. (a) The computational geometry of the stenosed model; (b) five-layer arterial wall with the thickness of each layer illustrated in the parentheses; (c) flow waveform at the inlet. EGL: endothelial glycocalyx layer; IEL: internal elastic lamina; *D*: inner diameter.

As a basic geometrical model, the arterial segment concerned was simplified as a straight axisymmetric cylinder. The inner diameter of model (*D*) and the wall thickness of the artery (*t*) are chosen to be *D* = 3.7 mm and *t* = 0.34 mm [13, 14]. The wall of the arterial segment was modeled as a five-layer structure and the thickness of each wall layer is shown in Figure 1(b) [15, 16], where the ratio of intima and media thickness was chosen to be approximately 0.75 [17].

For the stenosed model, the variation of the vessel radius along the stenosis was described using a cosine function and the reduction in the cross-sectional area of the lumen was set as 75% [18].

### 2.2. Governing Equations

#### 2.2.1. Lumen

The flow simulation in the lumen of the arterial segment was based on the incompressible Navier-Stokes and continuity equations:

(1) $$\begin{array}{c} \rho \left(\frac{\partial u}{\partial t}+u\cdot \nabla u\right)+\nabla p-\mu \Delta u=0,\\ \nabla \cdot u=0, \end{array}$$

where **u** and *p* represent, respectively, the fluid velocity vector and the pressure. *ρ* and *μ* are the density and viscosity of blood (*ρ* = 1050 kg·m^−3^, *μ* = 3.45 × 10^−3^ kg·m^−1^ s^−1^).

The mass transport of contrast agent Gd-DTPA in the flowing blood can be described by

(2) $$\begin{array}{c} \frac{\partial c}{\partial t}+u\cdot \nabla c-D_{G}\Delta c=0, \end{array}$$

where *c* is the concentration of Gd-DTPA, and *D*_*G*_ is the diffusion coefficient of Gd-DTPA in blood. At 37°C, when assuming that the radius of Gd-DTPA (*r*) is 1 nm [19], *D*_*G*_ can be calculated as 6.5847 × 10^−11^ m^2^ s^−1^ from the following Stokes-Einstein equation [20]:

(3) $$\begin{array}{c} D_{G}=\frac{kT}{6\pi \mu r}, \end{array}$$

where *k* is Boltzmann constant, and *T* is the absolute temperature.

#### 2.2.2. Arterial Wall Layers

The transmural flow across the arterial wall can be described by the Brinkman equation as follows [16]:

(4) $$\begin{array}{c} \frac{\rho }{\varepsilon_{l}}\frac{\partial u_{l}}{\partial t}+\frac{\mu_{l}}{K_{l}}u_{l}=-\nabla p_{l}+\frac{\mu_{l}}{\varepsilon_{l}}\nabla^{2}u_{l}, \end{array}$$

(5) $$\begin{array}{c} \nabla \cdot u_{l}=0, \end{array}$$

where **u**_*l*_ and *p*_*l*_ represent, respectively, the superficial velocity vector and the pressure based on the volume averaged method. *ε*_*l*_ and *K*_*l*_ are the porosity and the hydraulic permeability of the wall layer concerned. For all layers, the viscosity of plasma *μ*_*l*_ was assumed to be 0.72 × 10^−3^ kg m^−1^ s^−1^ [16].

The transport of Gd-DTPA across the arterial layers was modeled by the following equation [16]:

(6) $$\begin{array}{c} \frac{\partial c_{l}}{\partial t}+\left(1-\sigma_{l}\right)u_{l}\cdot \nabla c_{l}-D_{l}\Delta c_{l}=0, \end{array}$$

where *c*_*l*_ is the superficial concentration of Gd-DTPA; *D*_*l*_ is the effective diffusivity of Gd-DTPA in the arterial layers; *σ*_*l*_ is the filtration reflection coefficient.

### 2.3. Parameters

In order to solve (4)~(6), we have to acquire the values of the 4 parameters in the equations, namely, the porosity (*ε*_*l*_), the hydraulic permeability (*K*_*l*_), the effective diffusivity of Gd-DTPA (*D*_*l*_) and the filtration reflection coefficient (*σ*_*l*_) for each layer of the arterial wall. However, because it is hard to measure the parameters, the transport parameters are scarce. In the present study, most of the parameters were obtained from theoretical models. The calculation of the parameters is presented in Appendix. The parameters used are summarized in Table 1.

### 2.4. Boundary Conditions

As shown in Figure 1(a), flow transport equations ((1), (4)~(5)) are subject to the following boundary conditions.

 
*BC-A* at the inlet of the lumen of the arterial segment, a time-dependent fully developed (parabolic) inlet flow velocity profile was used for the pulsatile flow simulation (Figure 1(c)) [21]. The mean velocity was chosen to be 0.24 m s^−1^ [22].
 
*BC-B* the pressure at the outlet boundary of the artery lumen was set at 100 mm Hg.
 
*BC-C* at the media-adventitia interface, a constant pressure boundary condition with 30 mm Hg was employed.
 
*BC-D* symmetric conditions were set at the axis of symmetry.
 
*BC-E* no viscous flow was set on both the axial ends of the arterial wall.

The boundary conditions for the mass transport equations ((2) and (6)) are as follows.

 
*BC-1*: the injection of the contrast agent at the inlet was described by a gamma-variate function [12]:
  (7) $$\begin{array}{c} c\left(t,z=0\right)=\left\{\begin{array}{cc} a{\left(t-t_{0}\right)}^{b}e^{-g\left(t-t_{0}\right)}, & t>t_{0}\\ 0, & t\leq t_{0}, \end{array}\right. \end{array}$$
 where *a* = 1.013 × 10^−3^, *b* = 2.142, and *g* = 0.454 s^−1^. *t*_0_ was set to 3 s, which meant that the bolus of contrast was injected after the periodicity of the flow field was completely developed.
 
*BC-2*: for other boundaries, zero concentration gradient was assumed.

### 2.5. Computation Procedures

The numerical simulations were carried out using a validated finite element algorithm COMSOL Multiphysics. Three full cardiac cycles (3 s) simulation of the pulsatile flow with a time step of 4 ms were carried out to achieve a periodic flow independent of the initialization. Based on the initial velocity field, the mass transport equations were solved coupling with flow transport equations for 37 s with time step from 1 ms to 4 ms depending on the temporal variations of contrast concentration.

### 2.6. Quantification of Dispersion

The effect of the dispersion of the contrast agent bolus on the AIF can be described by convolving the estimated AIF with a vascular transport function (VTF) [4, 23]:

(8) $$\begin{array}{c} \text{AIF}\left(t-t_{0}\right)=\text{AIF}{\left(t\right)}_{\text{est}}⊗\text{VTF}\left(t-t_{0},z\right), \end{array}$$

where *t*_0_ is the bolus delay and *z* is distance to the inlet. Using the variance of the VTF around its mean, the degree of dispersion can be quantified:

(9) $$\begin{array}{c} \sigma_{\text{VTF}}^{2}\left(z\right)=\frac{\int_{0}^{\infty }{\left(t-\text{MVTT}\left(z\right)\right)}^{2}\cdot \text{VTF}\left(t,z\right)dt}{\int_{0}^{\infty }\text{VTF}\left(t,z\right)dt}, \end{array}$$

where the mean vascular transit time (MVTT) is given by the ratio of the first to the zeroth moment of the VTF and can be used to quantify the bolus delay:

(10) $$\begin{array}{c} \text{MVTT}\left(z\right)=\frac{\int_{0}^{\infty }t\cdot \text{VTF}\left(t,z\right)dt}{\int_{0}^{\infty }\text{VTF}\left(t,z\right)dt}. \end{array}$$

AIF_est_ is prescribed directly from the contrast agent concentration at the inlet ((7)  *c*(*t*, *z* = 0)) and AIF is computed as the area weighted average of the concentration of contrast agent on several lumen cross-sections between the inlet and the outlet perpendicular to the axial vessel direction (*c*(*t*, *z*)). To obtain the mean delay (MVTT) and the variance (*σ*_VTF_^2^), it is not necessary to calculate VTF firstly, since they can be calculated directly with the zeroth, first, and second integral moment of the concentration of the contrast agent [4]:

(11) $$\begin{array}{c} \sigma_{\text{VTF}}^{2}\left(z\right)=\frac{c^{\left(2\right)}\left(z\right)}{c^{\left(0\right)}\left(z\right)}-\frac{c^{\left(2\right)}\left(0\right)}{c^{\left(0\right)}\left(0\right)}+{\left[\frac{c^{\left(1\right)}\left(0\right)}{c^{\left(0\right)}\left(0\right)}\right]}^{2}-{\left[\frac{c^{\left(1\right)}\left(z\right)}{c^{\left(0\right)}\left(z\right)}\right]}^{2},\\ \text{MVTT}\left(z\right)=\frac{c^{\left(1\right)}\left(z\right)}{c^{\left(0\right)}\left(z\right)}-\frac{c^{\left(1\right)}\left(0\right)}{c^{\left(0\right)}\left(0\right)}. \end{array}$$

The moments of the concentration of the contrast agent are

(12) $$\begin{array}{c} c^{\left(n\right)}\left(z\right)=\overset{\infty }{\underset{0}{\int }}t^{n}\cdot c\left(t,z\right)dt. \end{array}$$

## 3. Results

### 3.1. Effects of the Arterial Transmural Transport on Contrast Agent Transport and the Bolus Dispersion

**Figure 2:**
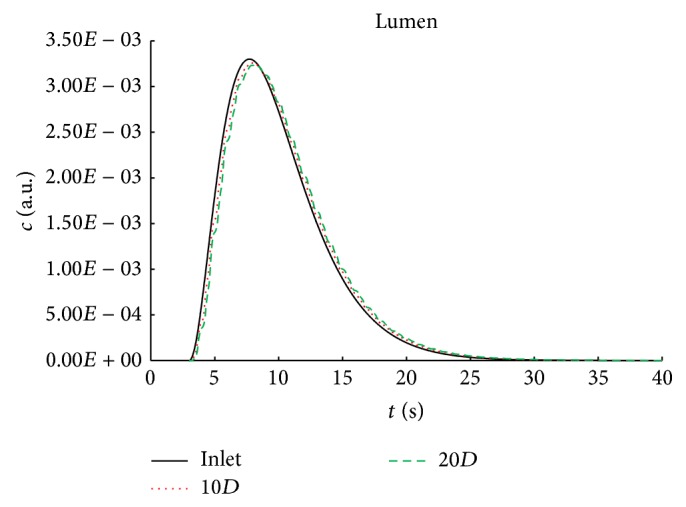
The concentration-time curves at inlet, 10*D*, and 20*D* in the model with arterial transmural transport.

**Figure 3:**
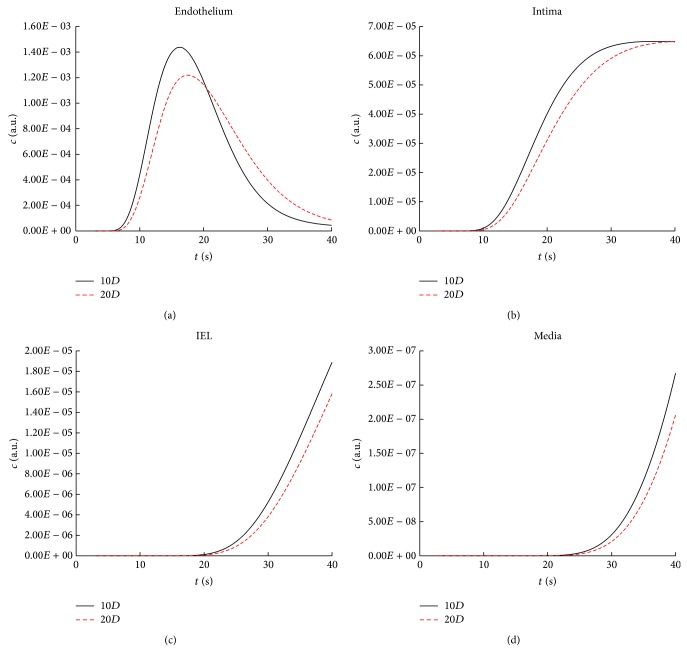
The concentration-time curves across the arterial wall at the axial direction of 10*D* and 20*D* in the model with arterial transmural transport.

To demonstrate the deformation of the concentration-time curve along the flow direction in the unstenosed model, the area weighted average of the concentration of contrast agent on cross-sections at axial direction of 10*D* and 20*D*  (*c*(*t*, *z*)) was calculated. As shown in the Figures 2 and 3, the concentration-time curve illustrates the characteristics of bolus delay with phase shift and bolus dispersion with relatively low maximal concentration and spread width profile. For instance, in the lumen of the artery, the maximal concentration at 20*D* shifts about 0.5 second and decreases 2%, when compared with that at the inlet (Figure 2). The delay and dispersion are enlarged in the arterial wall, resulting in that the maximal concentration at 20*D* in endothelial layer is about 1.3 second later and 18% lower than that at the 10*D* (Figure 3). In addition to the delay and dispersion in the axial direction, as demonstrated in Figure 2, the concentration of the contrast in the radial direction is much more decreased and spread. For instance, the maximal concentration of intima layer is approximately 20 times smaller than that of the endothelial layer and the time interval between the half maximal and maximal concentration at 20*D* at the intima layer is approximately 4 times longer than that at the endothelial layer (Figure 3).

**Figure 4:**
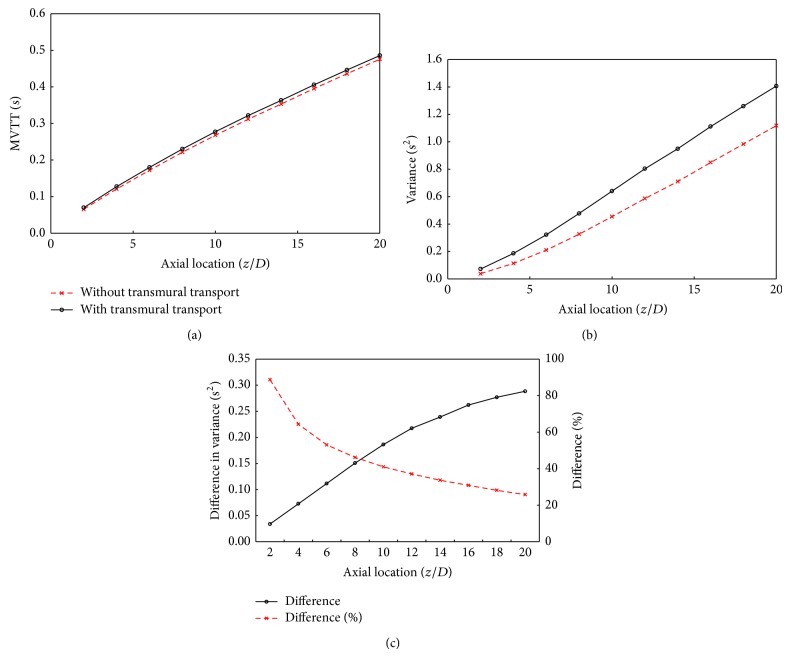
Comparison of the quantitative parameters of the bolus delay (MVTT) and dispersion (variance) in the unstenosed artery between the model with arterial transmural transport and without. (a) The mean vascular transit time (MVTT); (b) the variance of vascular transport function (variance); (c) the difference and percentage difference in variance.

The development of the bolus delay and dispersion in the unstenosed model are quantified by the mean vascular transit time (MVTT) and the variance of the VTF from (11). As illustrated in Figure 4(a), MVTT and the variance are increased almost linearly in relation to the distance to the inlet, which reflects consistently the concentration-time curve in Figure 2. When compared to the simulation with contrast agent transport only in the lumen, the arterial transmural transport would lead to slight increase in the MVTT and small increase in the variance (Figures 4(a)-4(b)) resulting in only increase of 0.186 s^2^ at 10*D* and 0.288 s^2^ at 20*D*. The quantitative difference in variance in Figure 4(c) further demonstrates that although the increase in the variance would increase gradually in relation to the distance to the inlet, the increase ratio would decrease sharply resulting in approximately 25% at 20*D*.

### 3.2. Effects of Endothelium on Contrast Agent Transport and the Bolus Dispersion

When the endothelium is damaged in diseased condition, the transport of the contrast agent may be affected and hence the bolus dispersion. To investigate the role of endothelium in the transport of the contrast agent, the endothelium was assumed to be totally damaged and the four transport parameters of the damaged endothelium and EGL were simplified to be similar to that of the intima. In addition, the transport parameters of other layers were consistent with that of the model with intact endothelium.

**Figure 5:**
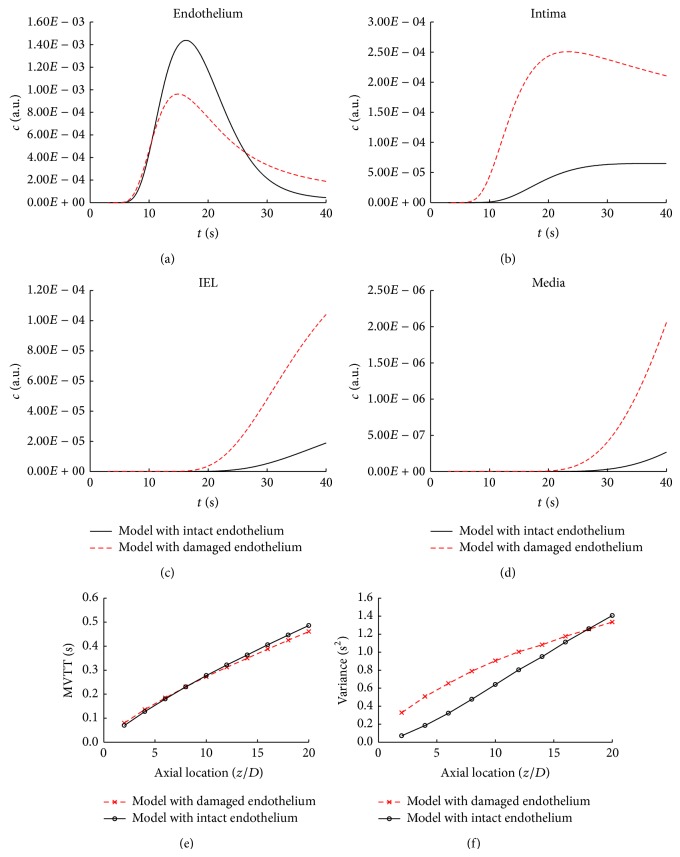
Effects of endothelium on contrast agent transport and the bolus dispersion in the unstenosed model. (a–d) Comparison of the concentration-time curve across the arterial wall between the damaged endothelium model and the intact one at the axial direction of 10*D*. (e-f) Comparison of the quantitative parameters of the bolus delay (MVTT) and dispersion (variance) between the damaged endothelium model and the intact one.

Figures 5(a)–5(d) demonstrate the concentration-time curve of the damaged endothelial model in the four arterial layers. As evident from these figures, due to the damage of the endothelium, the contrast agent is sharply accumulated in the arterial wall.

As illustrated in Figures 5(e)-5(f), the difference in the MVTT between the damaged endothelial model and intact one is slight and the difference in the variance between the two models decreases along the axial direction resulting in almost no difference at the outlet. These results indicate that the damage to the integrity of endothelium would have a small effect on the bolus delay and dispersion.

### 3.3. Effects of Disturbed Flow on Contrast Agent Transport and the Bolus Dispersion

Until now, the numerical simulations have been carried out only for a simplified straight axisymmetric blood vessel. However, the geometry of the physiological blood vessel is much more complex with such characteristics as branching, twisting, taper, and curvature, which would lead to far more than parabolic flow profile in the simplified model but very complicated flow patterns [24, 25]. One common flow pattern developed in the arterial system is the disturbed flow, which is inclined to occur in locations such as aneurysm, stenosed artery, the inner wall of curved segments, and the outer walls of arterial bifurcations [26, 27]. To further investigate the effects of arterial transmural transport on the bolus delay and dispersion under disturbed flow, contrast agent transport in the stenosed model was calculated.

**Figure 6:**
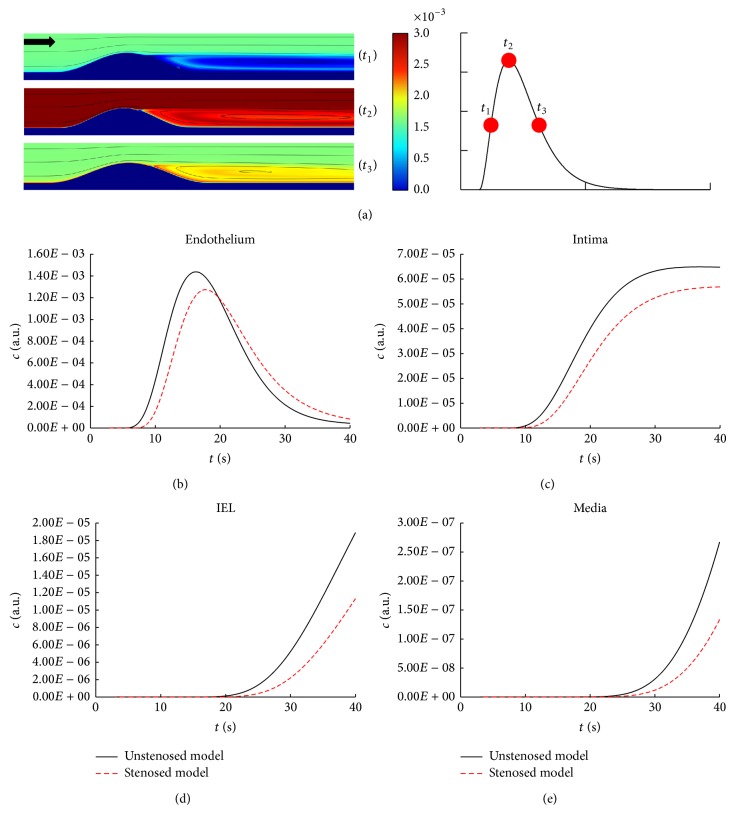
Effects of disturbed flow on contrast agent transport. (a) Concentration distribution in the stenosed region at three time points of the contrast injection at the inlet. *t*_1_ is the moment with half maximal concentration in the increasing phase; *t*_2_ is the moment with maximal concentration; *t*_3_ is the moment with half maximal concentration in the decreasing phase. (b–e) Comparison of the concentration-time curve across the arterial wall between the unstenosed model and the stenosed one at the axial direction of 10*D*.

Figure 6(a) shows the concentration distribution in the stenosed region at different moments of the contrast injection. It can be observed that the concentration in the disturbed flow region after the stenosis is quite uneven and significantly later than that of the regions of high flow.

Figures 6(b)–6(e) demonstrate the concentration-time curve in the four arterial layers at the disturbed flow region (10*D*). The concentration in the disturbed flow model decreases and spreads much more significantly than that in the straight model at the same axial location.

**Figure 7:**
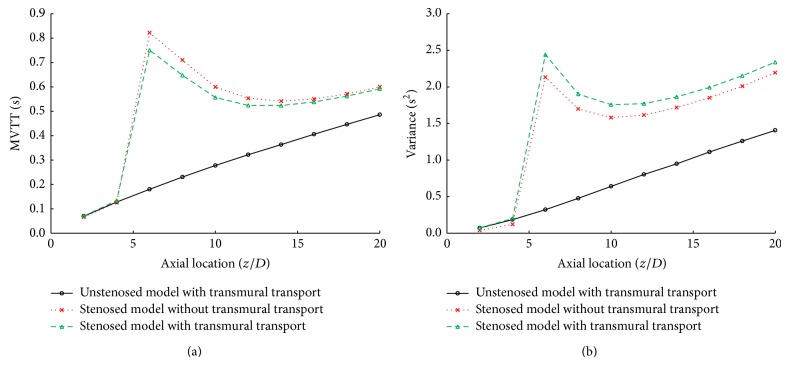
Comparison of the quantitative parameters of the bolus delay (MVTT) and dispersion (variance) in the stenosed artery between the model with arterial transmural transport and without. (a) The mean vascular transit time (MVTT); (b) the variance of vascular transport function (variance).

In the stenosed model the influence of the disturbed flow on the bolus delay and dispersion was observed immediately behind the stenosis (Figure 7). After the initial significant increase in the MVTT and variance behind the stenosis a reduction in the MVTT and variance follows in the disturbed flow zone. The arterial transmural transport would also affect the bolus delay and dispersion by decreasing the MVTT and increasing the variance. However, the effect of the arterial transmural transport on the MVTT and the variance is much less than that of the disturbed flow caused by the stenosis.

## 4. Discussion

One unreal assumption of DSC-MRI is that the arteries are impermeable to contrast agent. In the present study, we numerically coupled contrast agent transport in the arterial wall with that in arterial lumen to investigate whether the unreal assumption is valid for the estimation of the delay and dispersion of the contrast agent bolus. The results obtained reveal that the arterial transmural transport would slightly affect the bolus delay qualified by MVTT and moderately increase the bolus delay qualified by the variance. The MVTT and the variance induced by the arterial transmural transport are much less than that by the disturbed flow after the stenosis. In addition, although the integrity of endothelium can significantly affect the accumulation of contrast agent in the arterial wall, it has small effects on the bolus delay and dispersion.

The bolus dispersion would lead to a systematic blood flow underestimation. Graafen et al. simulated the dispersion in coronary arteries using a computational fluid dynamics approach and demonstrated that the variance between 1.0 and 2.5 s^2^ would lead to the myocardial blood flow underestimation between about 6% and 10% [11]. The present study demonstrated that the variance in both the stenosed and the unstenosed model is less than 2.5 s^2^ and the variance caused by the arterial transmural transport is less than 0.5 s^2^, which indicated that the myocardial blood flow error induced by the arterial transmural transport may be well below 10%.

As the transport of contrast agent is mainly governed by the convective transport in the lumen and the diffusion transport in lumen and the arterial wall, the local flow pattern, the flow rate, and the arterial transmural transport would affect the degree of bolus dispersion. Among the three factors, the arterial transmural transport may be the least important one, since the present simulation indicated that the disturbed flow produced much more dispersion of the bolus than the arterial transmural transport. However, the contribution of local flow pattern and the flow rate to the dispersion is comparable. Our simulations demonstrated the disturbed flow induced by the stenosis would significantly increase the bolus dispersion. In contrast, Graafen et al. indicated that stenosis in the coronary arterial model leads to a reduction of dispersion [11]. Calamante et al. found that for the two patients with similar degree carotid stenosis, only one of them had larger dispersion, while the other was found to produce a dispersion of the bolus similar to that found in the normal subjects [10]. These studies and our results indicated that, to achieve an accurate estimation of the dispersion, the simulations with patient-specific geometrical and boundary layer model should be performed and the effects of the arterial transmural transport may be neglected in practice.

This study revealed that local flow pattern caused by the stenosis would significantly affect the local contrast agent transport and hence the local bolus dispersion, which is constant with mass transport simulations based on the patient-specific model. For instance, Liu et al. demonstrated that the disturbed flow would significantly hinder the mass transport in the human aorta and computational studies indicated that the oxygen transport was significantly low in the outer wall of carotid artery where disturbed flow developed [28, 29]. Due to the fact that flow patterns are quite complex in the arterial segments such as the aneurysm, stenosed artery, the inner wall of curved segments, and the outer walls of arterial bifurcations [26, 27], the present results combining previous studies indicated that the arterial input function may be estimated beyond these regions to avoid large bolus dispersion [12].

In this pilot study, the accuracy of the results may be reduced by the simplifications of the transport parameters, the blood rheological properties, and the geometries.

Due to the scarcity of the transport parameters, most of the parameters were obtained from theoretical models. The estimated parameters, especially the diffusivity of the contrast agent, would affect the simulation results. The diffusivity used in the present study (6.5847 × 10^−11^ m^2^ s^−1^) is lower than the estimated diffusion coefficient of contrast agent in the previous studies (5.5 × 10^−10^ m^2^ s^−1^ and 1.5 × 10^−10 ^m^2^ s^−1^) [11, 12]. The lower diffusion coefficient in the lumen would lead to an increase of the dispersion due to a smaller exchange of contrast agent particles perpendicular to the flow direction. However, the lower diffusion coefficient in the arterial wall reduces the arterial transmural transport and hence decreases the dispersion in the lumen, which is shown in the present result that when compared to the damaged endothelial model the normal one with relatively low diffusivity leads to a small dispersion.

The blood in the present study was simplified as Newtonian fluid and the non-Newtonian rheological properties such as shear thinning nature of the blood were neglected. It is reported that the shear thinning non-Newtonian nature of blood could slightly reduce oxygen flux (similar micromolecule as contrast agent) in most regions of the arteries, and this effect became much more apparent in areas with disturbed flow [29]. Therefore, it may be valid to assume the blood Newtonian fluid to estimate the bolus dispersion in most regions of the model However, the assumption of Newtonian fluid may lead to an underestimation of dispersion in the disturbed flow region.

Another limitation of the present study is that all simulations were performed on simplified geometries. The geometry of the physiological blood vessel is very complex with such characteristics as branching, twisting, taper, and curvature, which would lead to complicated flow patterns. Therefore, it is necessary to use the realistic geometries to simulate the exact dispersion of the contrast agent bolus. As the main aim of the present study is not to estimate the exact dispersion errors in an individual patient, the results obtained from the typical parabolic flow and disturbed flow can still shed some light on the effects of arterial transmural transport of the contrast agent on the bolus delay and dispersion.

## 5. Conclusion

The arterial transmural transport would slightly affect the bolus delay and small increase the bolus dispersion, which may have small effects on the estimation of the blood flow estimation. In comparison with disturbed flow induced by the presence of stenosis, the arterial transmural transport plays a less important role in the bolus delay. Although the assumption of impermeable arterial wall of DSC-MRI is unreal, it may still be a good simplification for the estimation of the delay and dispersion of the contrast agent bolus.

## Figures and Tables

**Table 1:** Model parameters for each layer.

	EGL	Endothelium	Intima	IEL	Media
Hydraulic permeability (*K*, (m^2^))	6.0383 × 10^−18^	1.7383 × 10^−20^	4.2 × 10^−17^	8.4 × 10^−20^	6.09 × 10^−19^
Effective diffusivity (*D*_*e*_, (m^2^ s^−1^))	1.0128 × 10^−10^	3.0276 × 10^−13^	1.2219 × 10^−10^	2.4094 × 10^−13^	9.3392 × 10^−14^
Reflection coefficient (*σ*)	0.0555	0.1212	0.2514	0.2514	0.3617
Porosity (*ε*)	0.6735	0.0005	0.8025	0.002	0.258

## Appendix

### A. Parameters of the Model

#### A.1. Parameters of the EGL

The EGL layer of the arterial wall was assumed to be composed of symmetrical EGL fibers and leaky junctions of cells resulting from either dying or being in mitosis. According to the results by Liu et al. [15], the porosity and hydraulic permeability (*K*_egl_) of EGL fibers were obtained as 0.6735 and 6.0383 × 10^−18^ m^2^.

The total filtration reflection coefficient of the EGL layer is determined by

(A.1) $$\begin{array}{c} \sigma_{t\text{egl}}=\frac{K_{\text{egl}}\sigma_{\text{egl}}+K_{lj}\sigma_{lj}}{K_{\text{egl}}+K_{lj}}. \end{array}$$

The reflection coefficient of the fibers (*σ*_egl_) is given by [30]

(A.2) σegl=α+β2ln⁡(1+α/β)−α2β2−α3β−αβ−α4/4−α2/2β2−β4/4−ln⁡(β)−3/4,

where *α* is the ratio of the contrast agent Gd-DTPA radius (*r* = 1 nm) to the outer radius of the fluid annulus $R=3^{1/4}(2r_{f}+\Delta )/\sqrt{2\pi }$ (*α* = *r*/*R*) and *β* = *r*_*f*_/*R*. *r*_*f*_ is the fiber radius (6 nm). Δ is the space between fibers (8 nm).

In (A.1), the reflection coefficient (*σ*_*lj*_), the hydraulic permeability (*K*_*lj*_), and the effective diffusivity (*D*_*lj*_) of the leaky junctions are determined using the pore theory as follows [20]:

(A.3) σlj=1−23αlj21−αljFαlj−1−αlj23+23αlj−712αlj2,Fαlj=1.0−1.004αlj+0.418αlj3+0.210αlj4−0.1696αlj5,

where *α*_*lj*_ = *r*/*w* is the ratio of the radius of contrast agent Gd-DTPA to the half-width of the leaky junction (*w*), which is taken to be 20 nm [31]:

(A.4) $$\begin{array}{c} K_{lj}=K_{slj}\frac{A_{lj}}{S}, \end{array}$$

where *A*_*lj*_/*S* is the portion of leaky junctions on surface area *S* and *K*_*slj*_ is the hydraulic permeability of one leaky junction (*K*_*slj*_ = *w* ^2^/3). If we assumed that the leaky cell is located at the center of each periodic circular unit of radius *ξ* [31], then *A*_*lj*_/*S* can be determined by (2*πR*_cell_)(2*w*)/(*πξ* ^2^). Since the fraction of the leaky junctions, *ϕ*, which is defined as the ratio of the area of leaky cells to the area of all cells (*ϕ* = *R*_cell_^2^/*ξ* ^2^), can be determined from experimental results, (A.4) can be given by

(A.5) $$\begin{array}{c} K_{lj}=\frac{4}{3}\frac{w^{3}}{R_{\text{cell}}}\phi . \end{array}$$

The total effective diffusivity of the EGL is calculated from

(A.6) $$\begin{array}{c} D_{t\text{egl}}=D_{\text{egl}}+D_{lj}, \end{array}$$

where *D*_*lj*_ is the effective diffusivity of leaky junction:

(A.7) $$\begin{array}{c} D_{lj}=D_{slj}\frac{A_{lj}}{S}. \end{array}$$

The effective diffusivity of a single leaky junction is determined by [20]

(A.8) $$\begin{array}{c} D_{slj}=D_{\text{free}}\left(1-\alpha_{lj}\right)F\left(\alpha_{lj}\right), \end{array}$$

where *D*_free_ is the Gd-DTPA diffusivity in the free plasma, which is 3.1537 × 10^−11^ m^2^ s^−1^ at 37°C calculated from (3) with viscosity of *μ*_*l*_.

The effective diffusivity (*D*_egl_) for the EGL is obtained from the stochastic theory [32]:

(A.9) $$\begin{array}{c} D_{\text{egl}}=D_{\text{free}}\left[1-{\left(1-\varepsilon_{\text{egl}}\right)}^{0.5}\left(1+\frac{2r}{\pi^{0.5}r_{f}}\right)\right].\; \end{array}$$

After taking *R*_cell_ as 15 *μ*m and setting *ϕ* as 0.0005 [33], *σ*_*t*egl_ and *D*_*t*egl_ are determined as 0.0555 and 1.0128 × 10^−10^ m^2^ s^−1^, respectively.

#### A.2. Parameters of the Endothelium

Filtration flow can move through the endothelium via both normal junctions and leaky junctions of the endothelial cells. Therefore, the hydraulic permeability (*K*_end_) and the reflection coefficient (*σ*_end_) of the endothelium are given by

(A.10) $$\begin{array}{c} K_{\text{end}}=K_{lj}+K_{nj},\\ \sigma_{\text{end}}=\frac{K_{nj}\sigma_{nj}+K_{lj}\sigma_{lj}}{K_{nj}+K_{lj}}. \end{array}$$

For the normal junctions, the hydraulic permeability

(A.11) $$\begin{array}{c} K_{nj}=L_{nj}\mu_{l}l_{\text{end}}, \end{array}$$

where *l*_end_ is the thickness of the endothelium and *L*_*nj*_ = 1.576 × 10^−9^ m s^−1^ mm Hg^−1^ according to the experimental results [34].

The reflection coefficient of the normal junction is defined as

(A.12) σnj=163αnj2−203αnj3+73αnj4−169αnj21−αnj2Fαnj,Fαnj=21−αnj2−1−αnj4 ×1−2.1αnj+2.09αnj3−0.95αnj5.

*D*_*nj*_ is the Gd-DTPA diffusivity in the normal junctions, which can be determined as 3.02 × 10^−13^ m^2^ s^−1^ according to the radius of Gd-DTPA [35]. From (A.10) and (A.7), the hydraulic permeability, the reflection coefficient, and the effective diffusivity of the endothelium can be calculated as 1.7383 × 10^−20^ m^2^, 0.1212, and 3.0276 × 10^−13^ m^2^ s^−1^, respectively.

#### A.3. Parameters of the Intima

Using the results from Dabagh et al., the porosity and hydraulic permeability (*K*_int⁡_) of the intima are assumed to be 0.8025 and 0.42 × 10^−16^ m^2^ [36]. Applying the same method by Liu et al. [15], the effective diffusivity and the reflection of the intima can be obtained as 1.2219 × 10^−10^ m^2^ s^−1^ and 0.2514, respectively.

#### A.4. Parameters of the IEL

The IEL is assumed to have a constant thickness with fenestral pores. It seems that the fenestral pores were filled with the same fiber matrix as intima [37, 38]. Thus we took the same properties of the intima for the IEL to calculate the parameters and assumed that the fenestral pores were approximated as cylinder pores with a radius (*r*_fen_) of 1.5 × 10^−7^ m [33]. The hydraulic permeability (*K*_IEL_), the reflection coefficient (*σ*_IEL_), and the effective diffusivity of IEL (*D*_IEL_) are then determined as follows:

(A.13) $$\begin{array}{c} K_{\text{IEL}}=K_{\mathrm{int}}\varepsilon_{\text{IEL}}, \end{array}$$

(A.14) $$\begin{array}{c} \sigma_{\text{IEL}}=\sigma_{\mathrm{int}}, \end{array}$$

(A.15) $$\begin{array}{c} D_{\text{IEL}}=D_{\text{fen}}\varepsilon_{\text{IEL}}, \end{array}$$

where *ε*_IEL_ is the porosity of the IEL (0.002) and *D*_fen_ is the effective diffusivity of the contrast agency in the fenestral pores, which is calculated from

(A.16) $$\begin{array}{c} D_{\text{fen}}=D_{\mathrm{int}}F\left(\alpha_{nj}\right), \end{array}$$

(A.17) Fαfen=1−2.1044αfen+2.08877αfen3 −0.94813αfen5−1.372αfen6 +3.87αfen8−4.19αfen9.

*α*_fen_ is the ratio of the radius of contrast agent Gd-DTPA to the radius of fenestral pores (*α*_fen_ = *r*/*r*_fen_). From (A.13) and (A.15), the hydraulic permeability and the effective diffusivity of the IEL can be calculated as 8.4 × 10^−20^ m^2^ and 2.4094 × 10^−13^ m^2^ s^−1^, respectively.

#### A.5. Parameters of the Media

The media are modeled as a porous medium composed of smooth muscle cells with a hydraulic permeability of *K*_*m*_, given as [39]

(A.18) $$\begin{array}{c} K_{m}=K_{pm}\frac{1-F}{1-F}, \end{array}$$

where *K*_*pm*_ is the permeability of the porous medium and *F* is the fractional void volume of smooth muscle cells in the media. *K*_*m*_ equals 6.09 × 10^−19^ m^2^ after taking *K*_*pm*_ and *F* as 1.43 × 10^−18^ m^2^ and 0.4.

The effective diffusivity of the media is obtained from the following equation:

(A.19) $$\begin{array}{c} D_{m}=D_{pm}\frac{1}{1-F}\cdot \frac{1}{f\left(F\right)}, \end{array}$$

where *D*_*pm*_ is

(A.20) $$\begin{array}{c} D_{pm}=D_{\text{free}}\exp\left[-{\left(1-\varepsilon_{pm}\right)}^{1/2}\left(1+\frac{r}{r_{pm}}\right)\right]. \end{array}$$

*ε*_*pm*_ is the porosity of the extracellular fibrous phase in the media (0.43), *r*_*pm*_ is the radius of fibers in the extracellular matrix (3.22 nm), and *f*(*F*) is expressed as

(A.21) fF=21−4/πF ×arctan1−4/πF1−4/πF+arctan4/πF1−4/πF −π2+1−4πF.

The reflection coefficient of the media is given by

(A.22) $$\begin{array}{c} \sigma_{m}={\left(1-\phi_{m}\right)}^{2}, \end{array}$$

where

(A.23) $$\begin{array}{c} \phi_{m}=\exp\left[-\left(1-\varepsilon_{pm}\right)\left(2\frac{r}{r_{pm}}+\frac{r^{2}}{r_{pm}^{2}}\right)\right]\left(1-F\right). \end{array}$$

The porosity of the media is calculated as

(A.24) $$\begin{array}{c} \varepsilon_{m}=\varepsilon_{pm}\left(1-F\right). \end{array}$$

Thus, *D*_*m*_, *σ*_*m*_, and *ε*_*m*_ are determined as 9.3392 × 10^−11^ m^2^ s^−1^, 0.3617, and 0.258, respectively.
